# Prevalence of asthma and other allergic conditions in Colombia 2009–2010: a cross-sectional study

**DOI:** 10.1186/1471-2466-12-17

**Published:** 2012-05-02

**Authors:** Rodolfo J Dennis, Luis Caraballo, Elizabeth García, María X Rojas, Martín A Rondon, Adriana Pérez, Gustavo Aristizabal, Augusto Peñaranda, Ana M Barragan, Velky Ahumada, Silvia Jimenez

**Affiliations:** 1 Research Department, Fundación Cardioinfantil – Instituto de Cardiología, Carrera 13 B N° 163-85. Torre A, tercer piso, Bogota, Colombia; 2 Institute for Immunological Research, University of Cartagena, 6 No. 36-100, Cartagena, Colombia; 3 Fundación Santa Fe de Bogotá, Avenida 9 N° 117-120, second floor, Bogotá, Colombia; 4 Department of Clinical Epidemiology and Biostatistics, School of Medicine. Pontificia Universidad Javeriana, Carrera 7 N° 40-62, second floor, Bogotá, Colombia; 5 The University of Texas Health Science Center at Houston, School of Public Health, Division of Biostatistics, 1616 Guadalupe Street Suite 6.300, Austin, TX, 78701, USA; 6 Michael & Susan Dell Center for Healthy Living, 1616 Guadalupe Street Suite 6.300, Austin, TX, 78701, USA; 7 Pediatrics Department, Universidad El Bosque, Carrera 7 B Bis No. 132 – 11, Bogotá, Colombia

## Abstract

**Background:**

While it is suggested that the prevalence of asthma in developed countries may have stabilized, this is not clear in currently developing countries. Current available information for both adults and children simultaneously on the burden and impact of allergic conditions in Colombia and in many Latin American countries is limited. The objectives of this study were to estimate the prevalence for asthma, allergic rhinitis (AR), atopic eczema (AE), and atopy in six colombian cities; to quantify costs to the patient and her/his family; and to determine levels of Immunoglobulin E (IgE) in asthmatic and healthy subjects.

**Methods:**

We conducted a cross-sectional, population-based study in six cities during the academic year 2009–2010. We used a school-based design for subjects between 5–17 years old. We carried out a community-based strategy for subjects between 1–4 years old and adults between 18–59 years old. Serum samples for total and antigen-specific (IgE) levels were collected using a population-based, nested, case–control design.

**Results:**

We obtained information on 5978 subjects. The largest sample of subjects was collected in Bogotá (2392). The current prevalence of asthma symptoms was 12% (95% CI, 10.5-13.7), with 43% (95% CI, 36.3-49.2) reporting having required an emergency department visit or hospitalization in the past 12 months. Physician diagnosed asthma was 7% (95% CI, 6.1-8.0). The current prevalence of AR symptoms was 32% (95% CI, 29.5-33.9), and of AE symptoms was 14% (95% CI, 12.5-15.3). We collected blood samples from 855 subjects; 60.2% of asthmatics and 40.6% of controls could be classified as atopic.

**Conclusions:**

In Colombia, symptom prevalence for asthma, AR and AE, as well as levels of atopy, are substantial. Specifically for asthma, symptom severity and absence from work or study due to symptoms are important. These primary care sensitive conditions remain an unmet public health burden in developing countries such as Colombia.

## Background

There is data from epidemiological studies, mostly from middle and high income countries, that suggests the disease burden due to asthma may have stabilized or even decreased [1]‐[3]. It is not clear, however, if the same tendency would be observed in low to middle income countries, or by that matter, for allergic rhinitis (AR) and atopic eczema (AE) as well [4]‐[6]. Additionally, “new” associations, potentially causal, may keep disease burden increasing in different regions [7]‐[13].

Given the potentially inaccurate inferences when extrapolating research findings from different countries, especially on allergic conditions where the “drivers” of risk may be substantially different and vary dramatically among regions of the same country [14], it is crucial to have national estimates of epidemiological trends in these conditions. This is particularly important in Latin America, where the diversity of environmental conditions is high [15]. Colombia is a country of over 41 million inhabitants, predominantly urban (72%), where about 30% live in the four largest cities (Bogota, Medellin, Cali and Barranquilla). Similar to other countries in the Andean region (Venezuela, Ecuador, Peru, Bolivia), Colombia is experimenting changes consistent with a society in transition, with an aging population, where the main causes for death are chronic diseases. Colombia´s GIP, life expectancy, mortality rates and other basic health indicators are closer to those of Peru and Ecuador [16].

For Colombia, while previous research on asthma and other allergic conditions can serve as a good base for comparisons [5,6,17]‐[19], there is limited information available for both adults and children simultaneously on the current burden and impact of allergic conditions. This is the case as well for many Latin American countries, and certainly for those in the Andean and Caribbean regions.

The objectives of this study were to estimate the current prevalence of asthma, AR, AE and atopy in six colombian cities; to quantify costs to the patient and her/his family; and to determine levels of Immunoglobulin E (IgE) in asthmatic and healthy subjects.

## Methods

### Study design

We conducted a cross-sectional, population-based survey with an ancillary nested case–control study, in six Colombian cities: Barranquilla, Bogotá, Bucaramanga, Cali, Medellin, and San Andrés Island. Inclusion criteria were men or women aged 1–59 years old. Exclusion criteria included: (i) people confined in acute care hospitals or institutions for the chronically ill or for the disabled at the time of the study, and (ii) people with an altered mental state, dementia, or mentally challenged, because of the difficulty in collecting and assuring validity of information. The protocol was approved by the Clinical Research Ethics Committee at Fundación Cardioinfantil-Instituto de Cardiología in Bogotá, Colombia (IORG0006438).

### Sampling frame and sampling strategy

The sampling strategy differed based on subject age. First, we used a school-based design with a multistage cluster sampling to select children and adolescents (5–17 years old) that included representative samples from both private and public schools. The 2007 Colombian census was used as the sampling frame for randomization [20]. Since enrollment information was not available from all schools, two multistage probabilistic sampling strategies were implemented. For those schools with enrollment information, a probability proportional to size without replacement was implemented using enrollment for the cumulative total method [21], within city strata, school type (private or public), and education level. For those schools without enrollment information, multistage probabilistic sampling was carried out using simple random selection within strata of city, school type and education level. Within a school, a grade level was randomly selected and then one class group was randomly selected. All children and adolescents within the selected class group were invited to participate. A self-administered questionnaire was given to adolescents (13–17 years old) at their school. Parents of children (5–12 years old) were interviewed at home by study personnel.

Second, we carried out a community-based strategy to select subjects 1–4 and 18–59 years old. The homes of the randomly selected children and adolescent from the school-based design were considered “index” dwellings. We used a systematic approach to select eligible and consenting neighboring households close to the “index” dwelling. We contacted different households as necessary to include about four randomly selected subjects, each one from different households, for each “index” dwelling.

Third, we implemented a nested, case–control design based on asthma symptoms within this population-based study to assess the frequency of atopy. We defined a “case” as any subjects who answered yes to having asthma symptoms and a “control” as any subject who (i) reported not having had symptoms of asthma, allergic rhinitis, or atopic eczema in the last year; *and* (ii) reported no previous medical diagnosis of asthma, allergic rhinitis or atopic eczema. Additional consent to donate a blood sample of 3–10 mL was asked to all identified cases and controls. Cases and controls were frequency-matched by age group, gender, and city.

### Sample size

Sample size calculations were based on the Colombian population estimated from the 2005 national census and the estimated prevalence for each of the three diseases in a previous study in Colombia [17]. A sampling error between 1%-2% and a type I error of 0.05 was used. We estimated that 6,000 subjects proportionally allocated by population number in all six cities could give sufficient precision for nationwide estimates. Similarly, it was calculated that close to 10% of the surveyed subjects would be eligible for blood sampling for allergen-specific IgE antibody (sIgE) testing and total IgE (tIgE) determination [17].

### Questionnaire and data collection

Data collection took place during the academic year 2009–2010. The questionnaires were similar to those in a previous study [17]. Questions used were originally developed and validated by the International Study of Asthma and Allergy in Children initiative (ISAAC) [22]. We included a “does not know/does not respond” option in the questionnaire, and assumed for analysis as a “NO” answer. The calculations provided in the text and in the tables include this proportion of responses in the denominator of all prevalence estimates.

We added questions on absenteeism from school or work, health care consultants, and out-of-pocket costs. The definitions for current and past disease symptoms used for prevalence estimations were the following:

Asthma symptoms in the last year: Have you (or your child) had wheezing, or whistling in the chest in the past 12 months?

Past (lifetime) asthma symptoms: Have you (or your child) ever had wheezing or whistling in the chest at any time in the past?

Allergic rhinitis symptoms in the last year: In the past 12 months, have you (or your child) had a problem with sneezing or a running or blocked nose, when you (or your child) DID NOT have a cold or the flu?”

Past (lifetime) allergic rhinitis symptoms: Have you (or your child) ever had a problem with sneezing or a running or blocked nose, when you (or your child) DID NOT have a cold or the flu?”

Atopic eczema symptoms in the last year: “Have you (or your child) had this itchy rash at any time in the past 12 months?” and answered positively to the question, “Has this itchy rash at any time affected any of the following places: the folds of the elbows, behind the knees, in front of the ankles, under the buttocks, or around the neck, ears or eyes?”. These questions were preceded by: “Have you (have your child) ever had a skin rash which was coming and going for at least six months?

Past (lifetime) atopic eczema symptoms: Have you (have your child) ever had a skin rash which was coming and going for at least six months?

The definitions for disease severity and previous medical diagnosis of the three conditions were identical to those previously used in other ISAAC studies and can be found at http://isaac.auckland.ac.nz/resources/tools.php?menu=tools1.

### Immunoglobulin E (IgE) analysis

Serum samples were shipped following cold-chain standards to the Institute for Immunological Research at the University of Cartagena where all laboratory analyses took place. For each sample, total IgE (tIgE) and allergen-specific IgE antibody (sIgE) assays against *Dermatophagoides pteronyssinus* and *Blomia tropicalis* were performed using ImmunoCap system (Phadia) and following the technical instructions of the manufacturer. Atopy was defined as (i) tIgE levels greater than 100 kU/l or (ii) sIgE to *Blomia tropicalis* or *Dermatophagoides pteronyssinus* equal or greater than 0.35 kUA/l.

### Statistical analysis

Statistical analyses were carried out at the Pontificia Universidad Javeriana in Bogotá and at the University of Texas Health Science Center at Houston. For the random sampling, weights (the inverse of the selection probability of each observation) were calculated to account for differential inclusion probabilities due to the complex design; for this, information was obtained on the number of enrolled students during the 2009–2010 academic years in combination with the Colombian census bureau projections for year 2009. For the total sample, in addition to the previous weights, post-stratification weight adjustments were calculated to ensure that city, age, and gender composition in the sample was the same as in the sampling frame and census projections. Prevalence estimates are adjusted by city, age and gender. All the analyses took the form of weighted statistics with their 95% confidence intervals using the Taylor series linearization method for variance estimation [23]. Weighted percentages are reported for categorical variables. For continuous variables, weighted medians and inter-quartile range are reported. The Wilcoxon rank-sum test and chi-square statistics were used for statistical inference in the nested case–control study.

## Results

We obtained information on 5978 subjects (Table 1). In the school-based phase, 40.9% of schools, randomly selected, participated. In the community-based phase, participation ranged from 40%-70% between cities. In the nested case–control study, 53.4% of eligible case and 15.5% of eligible controls participated. The frequency of the“does not know/does not respond” option varied, but was below 3% for all questions in the questionnaire.

**Table 1:** Distribution of Subjects According to Sex, Age, Education and City in Colombia (2009–2010, n = 5978)

**Variable**	**No.**	**%**^**a**^	**95% CI**^**b**^	
Gender				
Male	2370	48.52	46.45	50.60
Female	3608	51.48	49.40	53.55
Age, yr				
1 – 4	466	7.09	6.76	7.43
5 – 17	1486	24.83	22.56	27.10
18 – 59	4026	68.08	66.01	70.15
Education				
None	29	0.65	0.40	0.90
Elementary School	3582	55.96	53.99	57.92
Middle/High School	1515	29.07	27.12	31.02
Associate Degree	290	5.74	5.05	6.43
College	361	7.92	7.08	8.76
Other^c^	26	0.56	0.33	0.78
Missing	5	0.11	0.01	0.21
City				
Barranquilla	700	8.72	7.98	9.46
Bogotá	2392	54.00	52.00	56.00
Cali	1021	16.32	15.15	17.49
Bucaramanga	453	3.81	3.34	4.29
San Andrés	401	0.51	0.45	0.58
Medellín	1011	16.63	15.13	18.13

^a^ Weighted Percentage (city, age and gender distribution of the general population).

^b^ CI: Confidence Interval.

^c^ Other: Doctorate, Masters, Police Academy, Art School, Security Training, Technician, etc.

### Asthma

The current prevalence of asthma symptoms varied between cities and by age group, but tended to be similar between genders (Table 2). Overall, the prevalence was 12% (95% CI, 10.5-13.7). Variations by city and age group, but not by sex, were also found for the lifetime accumulated prevalence of asthma symptoms (Table 3); the overall lifetime accumulated prevalence of asthma symptoms was 23% (95% CI, 21.1-24.8). The overall prevalence of physician-diagnosed asthma (Table 4) was much lower than symptom-based prevalence (7%; 95% CI, 6.1-8.0). Disease severity was high among those reporting asthma symptoms in the past 12 months; the prevalence reporting night awakenings due to symptoms was 58% (95% CI, 50.3-65.3), out of which 29% (95% CI, 23.7-34.3) reported symptoms 1–3 nights per week in the previous 12 months. Additionally, 43% (95% CI, 36.3-49.2) of subjects with reported asthma symptoms also reported requiring an emergency department visit or hospitalization in the last year.

**Table 2:** Prevalence of Asthma Symptoms in the Last Year According to Age, Sex and City in Colombia (2009 – 2010, n = 5978)

**Variable**	**No.**	**Yes**	**%**^**a**^	**95% CI**^**b**^	
Age, yr					
1 - 4	466	90	18.98	15.18	22.78
5 – 17	1486	256	16.78	11.30	22.26
18 - 59	4026	401	9.68	8.69	10.66
Gender					
Male	2370	298	12.13	9.41	14.86
Female	3608	448	12.07	10.47	13.67
City					
Barranquilla	700	119	14.52	11.85	17.19
Bogotá	2392	281	11.35	9.43	13.27
Cali	1021	133	13.35	10.98	15.72
Bucaramanga	453	72	13.95	10.00	17.89
San Andrés	401	42	11.70	7.38	16.02
Medellín	1011	99	11.63	5.20	18.06
Overall ^c^	5978	747	12.10	10.54	13.66

^a^ Weighted Percentage (city, age and gender distribution of the general population).

^b^ CI: Confidence Interval.

^c^ Adjusted by age, sex and city.

**Table 3:** Lifetime Prevalence of Asthma Symptoms According to Age, Sex, and City in Colombia (2009–2010, n = 5978)

**Variable**	**No.**	**Yes**	**%**^**a**^	**95% CI**^**b**^	
Age, yr					
1 - 4	466	175	36.51	31.83	41.19
5 – 17	1486	469	30.48	24.14	36.81
18 - 59	4026	780	18.83	17.52	20.14
Gender					
Male	2370	584	22.85	19.95	25.75
Female	3608	840	23.09	20.72	25.46
City					
Barranquilla	700	169	22.10	18.74	25.45
Bogotá	2392	527	21.71	19.04	24.38
Cali	1021	283	26.22	23.18	29.26
Bucaramanga	453	137	28.37	23.12	33.63
San Andrés	401	91	23.09	17.93	28.26
Medellín	1011	217	23.13	17.10	29.16
Overall ^c^	5978	1424	22.97	21.11	24.83

^a^ Weighted Percentage (city, age and gender distribution of the general population).

^b^ CI: Confidence Interval.

^c^ Adjusted by age, sex and city.

**Table 4:** Prevalence of Physician-Diagnosed Asthma According to Age, Sex and City in Colombia (2009–2010, n = 5978)

**Variable**	**No.**	**Yes**	**%**^**a**^	**95% CI**^**b**^	
Age, yr					
1 - 4	466	52	9.19	6.52	11.86
5 – 17	1486	149	8.59	5.50	11.68
18 - 59	4026	280	6.30	5.50	7.09
Gender					
Male	2370	201	6.98	5.51	8.45
Female	3608	280	7.16	5.92	8.39
City					
Barranquilla	700	76	9.98	7.57	12.39
Bogotá	2392	137	5.84	4.36	7.30
Cali	1021	86	8.66	6.51	10.82
Bucaramanga	453	53	10.29	7.10	13.49
San Andrés	401	46	11.28	7.34	15.21
Medellín	1011	83	7.11	5.19	9.02
Overall ^c^	5978	481	7.07	6.12	8.02

^a^ Weighted Percentage (city, age and gender distribution of the general population)

^b^ CI: Confidence Interval

^c^ Adjusted by age, sex and city

### Allergic rhinitis

There was a tendency in the current prevalence of AR symptoms to vary between cities and by age group (greatest in those 5–17 years old) (Table 5). Overall, the prevalence was 32% (95% CI, 29.5-33.9). The lifetime accumulated prevalence of AR symptoms was 38% (95% CI, 35.9-40.3). Among subjects reporting AR symptoms in the last year, the prevalence of physician diagnosed AR was 14% (95% CI, 12.4-16.1). The prevalence of allergic rhinoconjunctivitis during the previous year was 20% (95% CI, 19.0-21.0).

**Table 5:** Prevalence of Allergic Rhinitis Symptoms in the Last Year According to Age, Sex and City in Colombia (2009–2010, n = 5978)

**Variable**	**No.**	**Yes**	**%**^**a**^	**95% CI**^**b**^	
Age, yr					
1 - 4	466	145	30.44	25.96	34.91
5 – 17	1486	655	46.93	40.00	53.85
18 - 59	4026	1099	26.31	24.84	27.79
Gender					
Male	2370	749	31.07	27.63	34.52
Female	3608	1150	32.34	29.52	35.16
City					
Barranquilla	700	220	28.73	24.98	32.49
Bogotá	2392	758	32.61	29.16	36.05
Cali	1021	283	26.54	23.48	29.60
Bucaramanga	453	183	39.48	33.42	45.53
San Andrés	401	128	30.82	25.33	36.30
Medellín	1011	327	33.77	28.11	39.42
Overall ^c^	5978	1899	31.72	29.51	33.94

^a^ Weighted Percentage (city, age and gender distribution of the general population).

^b^ CI: Confidence Interval.

^c^ Adjusted by age, sex and city.

### Atopic eczema

The prevalence of AE symptoms in the last 12 months tended to vary little between cities or by age group, but tended to be larger for females (Table 6). Overall, the prevalence of AE in the last 12 months was 14% (95% CI, 12.5-15.3). The lifetime prevalence of AE symptoms was 24% (95% CI, 21.0-26.3), and the prevalence of physician diagnosed AE was 6% (95% CI, 4.8-6.3). The prevalence of those reporting one or more night awakenings per week in the previous year due to AE symptoms was 13% (95% CI, 9.3-15.9).

**Table 6:** **Prevalence of Atopic Eczema Symptoms**^**c**^**, in the Last Year According to Age, Sex and City in Colombia (2009–2010, n = 5978)**

**Variable**	**No.**	**Yes**	**%**^**a**^	**95% CI**^**b**^	
Age, yr					
1 - 4	466	102	19.53	15.76	23.30
5 – 17	1486	307	18.99	14.06	23.92
18 - 59	4026	478	11.45	10.39	12.51
Gender					
Male	2370	334	12.39	10.64	14.14
Female	3608	553	15.31	13.08	17.55
City					
Barranquilla	700	85	11.02	8.42	13.62
Bogotá	2392	335	13.60	11.25	15.95
Cali	1021	151	13.50	10.97	16.03
Bucaramanga	453	81	16.21	11.95	20.47
San Andrés	401	59	13.69	9.89	17.49
Medellín	1011	176	16.23	13.36	19.10
Overall ^d^	5978	887	13.89	12.45	15.33

^a^ Weighted Percentage (city, age and gender distribution of the general population).

^b^ CI: Confidence Interval.

^c^ Itchy rash during the last year affecting at least one of the characteristic places on body.

^d^ Adjusted by age, sex and city.

### Disease burden

Asthma was clearly the condition that generated the largest amount of out-of-pocket monthly expenditures (reported by 67% of asthmatic subjects). Asthma was also responsible for the highest frequency of reported absenteeism within the last six months from work or school, both for subjects and caregivers (Table 7); the median value of days away from school/work referred by asthma subjects was 4 days and 3 days for caregivers in the previous 6 months due to asthma. The most frequent cause for physician consultations within the past year was due to asthma symptoms (72%; 95% CI, 63.2-80.2).

**Table 7:** Disease Burden Associated with Asthma, Allergic Rhinitis, and Atopic Eczema in Colombia (2009–2010)

**Variables**	**Categories**	**%**^**a**^	**Median**^**b**^**(IQR**^**c**^**)**
Subjects reporting monthly out-of-pocket expenditures, past 6 months	Asthma	67.0	39,957 (80,756)
	Allergic Rhinitis	56.7	25,613 (43,098)
	Atopic Eczema	62.8	19,765 (47,577)
Subjects reporting days away from work/school, past 6 months	Asthma	55.7	3.98 (5.4)
	Allergic Rhinitis	32.9	2.78 (3.5)
	Atopic Eczema	17.7	4.52 (5.9)
Caregiver´s with days away from work/school, past 6 months	Asthma	43.4	2.96 (5.5)
	Allergic Rhinitis	26.8	2.29 (4.0)
	Atopic Eczema	13.0	4.74 (11.6)
Subjects reporting physician consultations, past 12 months	Asthma	71.7	n/a^d^
	Allergic Rhinitis	60.5	n/a^d^
	Atopic Eczema	64.1	n/a^d^

^a^ Weighted Percentage of those subjects with disease symptoms in the previous 12 months.

^b^ Colombian pesos of 2009.

^c^ IQR: Interquartile range.

^d^ n/a: not applicable.

### Atopy

We were able to collect blood samples from 855 subjects; 399 with asthma symptoms in the last year, and 456 controls. Table 8 shows that levels of tIgE and sIgE (against *Dermatophagoides pteronyssinus* and *Blomia tropicalis)* were higher among cases than controls. Sixty percent of cases and 41% of controls were classified as atopic. Subjects with asthma symptoms and with current symptoms of AR and AE had a prevalence of atopy of 63% (95%CI: 53%-72.5%).

**Table 8:** **Total IgE and Specific IgE Against** ***Blomia tropicalis*** **and** ***Dermatophagoides pteronyssinus*** **Levels by Case or Control Status in Colombia (2009–2010)**

**Description**	**Asthma Cases**	**Controls**	***P*-value**
	**n = 399**	**n = 456**	
Total IgE (kU/l), median (IQR^a^)	122.0 (340.80)	50.3 (121.00)	<0.0001^b^
Subjects above cutoff^c^, No. (%)	212 (53.13%)	149 (32.68%)	<0.0001^d^
Specific IgE BT (kUA/l), median (IQR^a^)	0.1 (4.11)	0.01 (0.15)	<0.0001^b^
Subjects above cutoff^e^, No. (%)	164 (41.10%)	89 (19.52%)	<0.0001^d^
Specific IgE DP (kUA/l), median (IQR^a^)	0.15 (12.48)	0.03 (0.14)	<0.0001^b^
Subjects above cutoff^e^, No. (%)	184 (46.12%)	95 (20.83%)	<0.0001^d^

^a^ IQR: Interquartile range.

^b^ Wilcoxon rank-sum test.

^c^ Cutoff level: Total IgE 100 kU/l.

^d^ Chi-square test.

^e^ Cutoff level: Specific IgE 0.35 kUA/l.

## Discussion

This cross-sectional study describes the prevalence of asthma, AR and AE symptoms in six Colombian cities, as well as their severity and burden. It provides as well the prevalence of atopy in asthmatic subjects, and in subjects without these conditions. The study is important because it is able to provide this information in subjects between 1 and 59 years of age. Additionally, this study is uniquely poised to generate vital surveillance information in Colombia, and increases the understanding of the public health burden due to these conditions in developing countries.

The current study used the same questions as the ISAAC initiative worldwide, and was conducted in the same cities in Colombia as a previous study from the same research group eleven years ago [17]. However, differences in age groups, methods and in data analysis, as well as in allergens used for sIgE, preclude a formal comparison between the two surveys, or with others conducted previously in Colombia and abroad. Thus, contrasts discussed here can only suggest potential tendencies over time. Our study suggests that the prevalence of symptoms of all three conditions is increasing in Colombia. This tendency towards an increase is similar to that found in ISAAC studies over an average of 8 years in the region; for the 13–14 age group, Latin America was one of the few regions in the world (with Africa) that had more centres increasing prevalence of all three diseases simultaneously [4]. The wide variations observed in this study in the prevalence of the three conditions, as well as those observed between cities and for the different age groups, have been found previously elsewhere [24], as well as for Latin American countries [25]. These differences suggest that local factors may dramatically alter the prevalence of these conditions, and they can include a wide number of both genetic and environmental characteristics acting simultaneously and synergistically. The study by Mallol et al [25] that included three cities in Colombia (Bogota, Cali, Barranquilla), did not find a positive associations between asthma and the socioeconomic variables studied (latitude, altitude, national gross income, poverty). The Isaac III study in Bogota, however, found that asthma symptoms were correlated with the presence of a cat in the home, higher maternal education, watching televison 1 to 2 hours per day, and the use of acetaminophen and antibiotics in the last year [18]. In that same study, rhinoconjunctivitis symptoms were associated with previous acetaminophen and antibiotic use, and higher maternal education and cesarean birth [26]. Additional risk factors in Latin America have also been postulated and described in detail by Cooper et al elsewhere [27,28].

The prevalence of current asthma symptoms (12%) seems to have increased in Colombia with respect to results obtained 11 years ago (10%) [16]. The age group where asthma symptoms where most frequent in both surveys is the 1–4 year old group. It is likely that some of these symptoms correspond to wheezing episodes associated with respiratory viral infections early in life [29]; recent evidence, however, also suggest that bronchial obstruction during acute respiratory infection in childhood is clearly associated with subsequent asthma, especially among school-aged children at risk for repeated asthma exacerbations [30], making it relevant to quantify and to include in epidemiological studies. In the current survey we found an increase in current asthma symptoms for those subjects 5–17 years of age. In the ISAAC I-III 13–14 age group, similar increases were found in Latin America for Mexico, Costa Rica, Panama, Chile, and Argentina [4].

Disease severity for asthma remains high when compared with results 11 years ago. This may be due to under-diagnosis of asthma; in both surveys, the frequency of physician diagnosis is very low when compared to symptom-based prevalence, especially for subjects below age 18 years of age, suggesting limited access to specialized health care in these age groups. Suboptimal asthma control can be another explanation for persistent disease severity, due to lack of general physician awareness on current guidelines on effective treatment strategies for asthma [31,32]. Disease burden to the patient and the caregiver was important in our study, with more of 60% of asthma patients reporting out-of-pocket expenses. Inappropriate ambulatory care of subjects with asthma can also be associated with increased hospitalization due to exacerbations; asthma is an ambulatory care sensitive condition where the need for hospitalization can be a marker of inappropriate health care [33]. Additionally, the mean number of days lost from work or school is our study is consistent with moderate persistent asthma, a stage of severity associated with substantial health care costs and hospitalizations in previous studies in the literature [34,35].

While the presence of asthma independent of increased IgE levels is not uncommon [36], the majority (60%) of subjects with asthma symptoms in the past year met the criteria for atopy in our study. This is lower than the frequency obtained in the previous study in 1999 (76%), but still high. The frequency of atopy may vary between regions and countries, which is expected because it depends on environmental and genetic factors [37]. There were statistically significant differences in atopy between cases and a control group in our study. In our population, the high frequency of atopy among controls may be explained in part because of exposure to helminths, mainly *Ascaris lumbricoides* infections, early in life [38]. Nematode exposure induces specific IgE responses and increases total IgE levels. In the tropics, permanent exposure to mite allergens and parasite infections during childhood may induce sIgE to cross-reacting allergens from mites and *Ascaris*[39,40].

The prevalence of AR symptoms also seems to have increased with respect to the previous study (from 23% to 32%) [17], but with a modest increase compared to a more recent survey in three Colombian cities that used the same ISAAC methodology during 2002 [41]. The larger increase in AR symptoms (than that seen for asthma symptoms) is in line with the ISAAC I-III comparisons in Latin America, where more centres had an increase for AR than for asthma [4]. With 28% current prevalence of rhinoconjunctivitis in children 5–17 years old (data not shown), Colombia would be among the countries in the region with the largest prevalence, as detected by ISAAC methodology [4,6,41]. Similarly, the prevalence of AE symptoms (14%) increased markedly in comparison to results obtained 11 years ago (4%). The current prevalence of AE symptoms has increased as well when compared with results obtained for Colombia during 2002 in the 6–7 and 13–14 year old population [5].

Our study has limitations. First, results may not be extrapolated to children not attending school or living in rural dwellings in Colombia. Second, the community-based strategy was not random and selection bias is possible if subjects willing to participate were also more likely to have one of the conditions under study, biasing results towards higher disease prevalence. While we do not have information on non-participants to evaluate this possibility, we used a systematic approach to select eligible and consenting neighboring households, and subjects within households were randomly selected. Third, our choice of controls to assess differences in atopy (excluding those with allergic rhinitis and atopic eczema) can be questioned; while such exclusions may have biased the prevalence of atopy in the control group below that in the source population of cases, we believe that exclusion of these two conditions provide a better estimate of the prevalence of atopy in the population sampled. Fourth, given that we used two allergens to diagnose atopy by sIgE (extracts from *D. pteronyssinus* and *B. tropicalis*), it is possible that the effects of cross reacting antibodies were more relevant. Differences of allergen sensitization between patients and controls could have been greater if we had included other allergens of interest, such as *Dermatophagoides farinae*, but financial limitations and the population-based design precluded this.

## Conclusions

Our results show that in contrast to more affluent countries [42]‐[44], these allergic conditions are a significant problem in urban cities in Colombia, that severity of asthma is substantial, and that levels of atopy are highly associated with asthma symptoms. Since the allergic component of asthma seems to be important, consideration should be given to its evaluation during diagnosis. Additionally, results of this work support the increasing evidence that the prevalence of asthma and other allergic diseases in Latin America is high [26], even though the climate and hygiene conditions are different than those of industrialized countries. Future research in the field should look for common risk factors acting at different times in developed and developing countries.

## Competing interests

All authors declare that they have no competing interests.

## Authors' contributions

RJD conceived the study and had primary responsibility for design, overall planning, interpretation, and drafted the manuscript. LC participated in the conception, design, interpretation, analysis of data, and drafting the manuscript. EG participated in the conception, design, interpretation, analysis of data, and revised the manuscript. MXR participated in the design, analysis of data and drafting the manuscript. MAR participated in the design of the study, helped with planning data collection, performed the statistical analysis and revised the manuscript. AP participated in the design of the study, overall planning of data collection, helped with statistical analysis and drafting the manuscript. GA participated in the design, interpretation and analysis of data, and revised the manuscript. AP participated in the design, interpretation of data and helped revise the manuscript. AM B participated in the design, coordinated data collection, helped with interpretation and data analysis and revised the manuscript. VA participated in data analysis, laboratory assays, participated in the interpretation of data and revising of the manuscript. SJ participated in data analysis, laboratory assays, the interpretation of data and revising of the manuscript. All authors read and approved the final manuscript.

## Pre-publication history

The pre-publication history for this paper can be accessed here:

http://www.biomedcentral.com/1471-2466/12/17/prepub
